# A Retrospective Study Evaluating the Feasibility and Outcomes of a Robotic-Assisted General Surgery Program in a Nontertiary General Hospital: Insights From Our First 100 Consecutive Cases

**DOI:** 10.7759/cureus.86318

**Published:** 2025-06-18

**Authors:** Alexis Terras, Georgios Tzikos, Adel Assiri, Mohammad Kasim, Rishi Banerjee, Alistair Myers, Yasser Mohsen, Alistair Slesser

**Affiliations:** 1 General Surgery Department, The Hillingdon Hospitals NHS Foundation Trust, London, GBR; 2 1st Propaedeutic Department of Surgery, AHEPA University Hospital of Thessaloniki, Thessaloniki, GRC

**Keywords:** first 100 experience, general surgery, learning curve, nontertiary hospital, robotic-assisted surgery (ras)

## Abstract

Introduction

Robotic-assisted surgery (RAS) has transformed minimally invasive surgery by enhancing precision and control. This study evaluates the feasibility and outcomes of introducing a RAS program at a nontertiary hospital, addressing resource and training challenges.

Materials and methods

A retrospective cohort analysis of 100 consecutive RAS procedures performed between December 2023 and September 2024 in the Colorectal Department at The Hillingdon Hospitals NHS Foundation Trust was conducted. Procedures utilized the Intuitive da Vinci Xi Surgical System and involved three senior colorectal consultants.

Results

Patients (median age: 59.0 years, 51 females) underwent 48 minor and 52 major procedures, with cholecystectomy (n = 35) and sigmoidectomy (n = 22) being most common. Median operative times were 88.5 minutes for minor (interquartile range (IQR): 47.3) and 263.0 minutes for major procedures (IQR: 148.8). Median hospital stays were zero days for minor and six days for major procedures. Complication rates were low, with three conversions to open surgery, one Clavien-Dindo grade 3b complication, and one 30-day mortality (major cases only).

Conclusions

The program demonstrated safety and favorable outcomes, even in its early phase, with effective adoption in a resource-limited, nontertiary setting. These results support the feasibility of implementing RAS programs in similar healthcare environments.

## Introduction

Robotic-assisted surgery (RAS) has significantly advanced the possibilities within minimally invasive surgery, allowing surgeons to achieve levels of precision and control that can be challenging with traditional methods. The United Kingdom first introduced RAS systems, such as AESOP and ZEUS, in the late 1990s, followed by the da Vinci system in 2000. Since its introduction, robotic surgery has expanded rapidly worldwide, with over 12 million procedures performed across 70 countries, including more than 1.8 million in 2022 alone [1]. 

While RAS was initially adopted in cardiac surgery, it quickly gained traction in urology, where it demonstrated considerable advantages in prostatectomy, such as reduced blood loss, shorter hospital stays, and improved resection margins [2]. Despite these promising outcomes, adoption of RAS across other surgical fields has been more gradual. This slower uptake can be attributed to the high costs of acquiring and maintaining robotic systems and the need for robust evidence to support their use across various healthcare settings [3]. In 2018, the Royal College of Surgeons highlighted the potential of RAS to improve surgical training, precision, and patient care in the UK [1]. However, implementation remains inconsistent, often dependent on local resources and expertise rather than a coordinated national strategy.

In particular, district general hospitals face specific challenges in adopting RAS, as they must carefully allocate limited resources and address training needs while serving broad patient populations. In these hospitals, conventional laparoscopic techniques have long provided minimally invasive options but are often restricted by nonwristed instruments, the reduced intuitive movement of trocars, and ergonomic strain for surgeons [4,5]. These constraints underscore the appeal of RAS in such settings, though financial and logistical barriers continue to hinder its broader integration. This study aims to evaluate the initial robotic-assisted general surgery program at The Hillingdon Hospitals NHS Foundation Trust, assessing the feasibility and primary outcomes of introducing a new robotic surgical system.

## Materials and methods

Study design

This retrospective cohort study was conducted from December 2023 to September 2024 in our department. All robotically assisted procedures were included in the analysis. The a priori inclusion criteria required patients to be over 18 years of age and to provide informed consent prior to surgery. The study received approval from the hospital’s ethics review board. All procedures were conducted by three senior colorectal surgery consultants, each having completed the required training and proctorship as stipulated by the Royal College of Surgeons guidelines. The robotic system utilized for all surgeries in this study was the Intuitive da Vinci Xi Surgical System (Intuitive Surgical, Sunnyvale, CA).

Data collection

Data collection was carried out by two junior resident investigators. All patient data were initially stored anonymously in a secure MS Excel (Microsoft Corporation, Redmond, Washington, United States) spreadsheet. To facilitate further analysis, a Statistical Analysis System (.sav) file was created for editing by the statistician using statistical software. The collected data included patient demographics (age and gender), anthropometric measurements (body mass index (BMI)), preoperative details (American Society of Anesthesiology (ASA) score and history of previous abdominal surgery), procedure characteristics (type of operation, urgency, ambulatory status, indication, and severity), intraoperative details (procedure duration, conversion rate, and intraoperative complications), and postoperative outcomes (total length of stay (LOS), intensive care unit (ICU) admission rate, ICU LOS, reoperation rate, major postoperative complications defined as Clavien-Dindo more than 3, readmission rate, and 30-day mortality). Finally, the operations were classified as minor and major based on the extent of surgical intervention, associated risks, and postoperative recovery. This classification was decided upon as an attempt to logically organize and interpret our mixed data. Cholecystectomy, hernia repair, and appendectomy were considered minor operations as they are typically less invasive, involve lower risk, have shorter operating times, and usually allow for same-day discharge. In contrast, major operations included large bowel resections, which are more complex, involve significant tissue dissection or organ resection, carry higher risks, and require longer recovery times.

Statistical analysis

The normality of the data distribution was assessed using the Shapiro-Wilk test when the sample size was ≤50, or the Kolmogorov-Smirnov test when the sample size was >50, respectively. For continuous variables, data were reported as means ± standard deviation (SD) if the data were normally distributed or as median and interquartile range (IQR) if normality was not assumed. Categorical variables were presented as percentages. Comparisons between means and medians for continuous data were made using the Student’s t-test or the Wilcoxon test, as appropriate. Differences in categorical data were analyzed using the Chi-square test. Statistical analyses were performed using the IBM SPSS Statistics for Windows, Version 25 (Released 2017; IBM Corp., Armonk, New York, United States). A p-value of <0.05 was considered statistically significant. All the figures were created by means of Microsoft Excel Professional Plus 2019.

## Results

Patient demographics and baseline characteristics

A total of 100 consecutive patients were included in this study. Patients’ median age was 59 years (IQR: 30.3), and the cohort consisted of 49 males (49%) and 51 females (51%), demonstrating a nearly even gender distribution. The mean BMI was 27.5 kg/m² (SD: 5.0). Regarding the ASA classification, the majority of patients (76 patients, 76%) had an ASA score of 2, indicating mild to moderate systemic disease. Approximately 25% of patients had a history of prior abdominal surgery. The severity of procedures was classified into minor (48 cases, 48%) and major (52 cases, 52%). Further sub-analysis revealed that patients in the major operation group were statistically older and predominantly male compared to those in the minor operation group. The baseline characteristics, categorized by procedure severity (minor vs. major), are summarized in Table 1.

**Table 1:** Baseline characteristics of patients, categorized by procedure severity ASA: American Society of Anesthesiology; BMI: body mass index *Data are presented as median (interquartile range). **Data have been presented as N (%). ***Data are presented as mean (standard deviation). p-value < 0.05 is considered significant

Severity of procedure	Minor	Major	p-value	t-value or Chi-square
Number of patients	48	52		
Age (years) *	50.0 (23.0)	69.0 (16.3)	<0.001	t-value: 4.044
Gender			0.003	Chi-square: 9.066
Male	16 (33.3%)	33 (63.3%)		
Female	32 (66.7%)	19 (36.5%)		
BMI (Kg/m^2^) **	27.9 (5.2)	27.0 (4.8)	0.491	t-value: 0.688
ASA score			0.001	Chi-square: 13.915
1	6	0		
2	39	37		
3	3	15		
Prior abdominal surgery			0.644	Chi-square: 0.214
Yes	13 (27.1%)	12 (23.1%)		
No	35 (72.9%)	40 (76.9%)		

Procedural characteristics

Among the procedures performed during this period, cholecystectomy was the most common (35 cases, 35%), followed by sigmoidectomy/high anterior resection (22 cases, 22%) and right colectomy (13 cases, 13%). Eight hernia repairs were conducted (8%), comprising seven inguinal hernia repairs (7%) and one primary midline hernia repair (1%). Other procedures included appendectomy, low and ultralow anterior resection, subtotal colectomy, abdominoperineal resection, and three other miscellaneous procedures, including a mesenteric lymph node biopsy, a ventral mesh rectopexy, and a completion proctectomy. The vast majority of cases (98%) were elective; 40% of cases were managed as day surgeries, while malignant disease was present in 42% of cases (Table 2).

**Table 2:** Procedures’ characteristics Y: yes; N: no The data has been represented as N (%)

Type of procedure	
Cholecystectomy	35 (35%)
Hernia (inguinal and primary midline)	8 (7 and 1) (8%)
Appendectomy	5 (5%)
Right colectomy	13 (13%)
Sigmoidectomy/high anterior resection	22 (22%)
Low and ultralow anterior resection	8 (8%)
Subtotal colectomy	3 (3%)
Abdominoperineal resection	3 (3%)
Other	3 (3%)
Elective surgery (Y/N)	98 (98%)/2 (2%)
Day surgery (Y/N)	40 (40%)/60 (60%)
Malignant disease (Y/N)	42 (42%)/58 (58%)

Intraoperative data

Median operative times were significantly longer for major procedures compared to minor ones, with times of 263.0 minutes (IQR: 148.8) for major procedures and 88.5 minutes (IQR: 47.3) for minor procedures (p < 0.001). Conversion to open surgery occurred in 5.8% of major cases, whereas no conversions were observed in minor cases (p = 0.085). The overall intraoperative complication rates were comparable, at 2% for minor procedures and 2.1% for major procedures, with no statistically significant difference (p = 0.337).

Postoperative outcomes

Postoperative outcomes varied significantly by procedural severity. For minor procedures, the median total LOS was zero days, while major procedures, as it was expected, had a significantly higher median LOS of six days. ICU LOS and admission rates differed significantly as well; no minor cases required ICU admission, whereas 57.7% of major cases did. Major complications were observed in 1.9% of cases in the major operation group (p = 0.008). Reoperation rates were 2.1% and 1.9% for minor and major procedures, respectively; readmission rates were 4.2% for minor procedures and 5.8% for major procedures. There was no statistically significant difference in readmission rates and the 30-day mortality rate (Table 3).

**Table 3:** Postoperative outcomes LOS: length of stay *The data have been presented as mean and standard deviation. **The data have been presented as percentages, while the numbers in the parentheses represent the number of patients. p-value < 0.05 is considered significant

Severity of procedure	Minor (n = 48)	Major (n = 52)	p-value
Total LOS (days) *	0.0 (0.0)	6.0 (4.0)	<0.001 (t-value: 8.270)
ICU LOS (days) *	0.0 (0.0)	1.0 (2.0)	<0.001 (t-value: 6.220)
ICU admission rate	0%	57.7%	<0.001 (Chi-square: 40.512)
Reoperation **	2.1% (1)	1.9% (1)	0.965 (Chi-square: 0.002)
Major complication **	0.0% (0)	1.9% (1)	0.008 (Chi-square: 6.947)
Readmission **	4.2% (2)	5.8% (3)	0.697 (Chi-square: 0.152)
30-day mortality **	0.0% (0)	1.9% (1)	0.330 (Chi-square: 0.951)

Subgroup analysis

Robotic Cholecystectomies

The subgroup analysis of robotic cholecystectomy cases revealed that all 35 procedures (35%) were performed electively. Of these patients, 30 were females (85.7%). The median hospital LOS was zero days, with only one patient requiring a stay of six days; the remaining five patients who required hospitalization had an LOS ranging between one and two days. The median patient age was 49.5 years (range: 24-79), and the median BMI was 27.9 kg/m² (range: 17.3-39.4). In terms of comorbidity classification, the majority of patients were classified as ASA 2 (n = 29, 82.9%).

**Figure 1:**
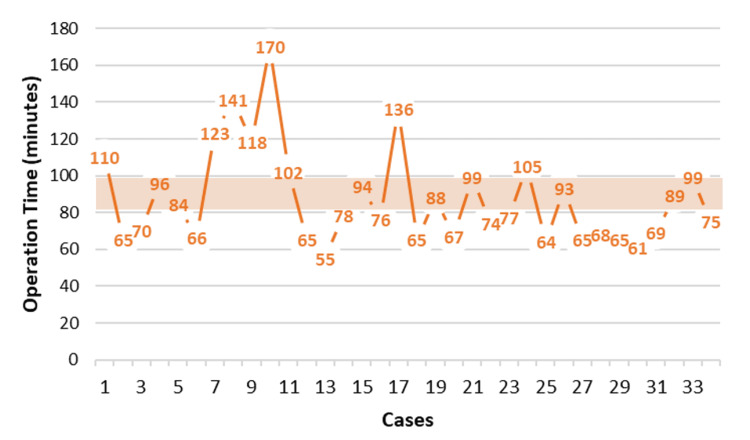
Evolution of intraoperative cholecystectomy time The shadowed area represents the average time reported in the literature [6]

The median operative time was 81 minutes, with an IQR of 37.8 minutes (range: 55-170 minutes). Notably, one outlier, a patient with chronic cholecystitis, significantly contributed to the upper range of operative times. The graph of intraoperative time evolution demonstrates this variability, indicating a generally consistent trend with occasional outliers (Figure 1).

Postoperative outcomes were favorable, with no reoperations or conversions to open procedures. One patient (2.9%) required readmission for pain control; however, imaging did not reveal any complications. Of the 35 cholecystectomy cases, 28 (80%) were successfully managed as ambulatory surgeries. Seven patients (20%) required hospitalization, three of whom had a history of prior abdominal surgery. The median BMI for these patients was higher (31.9 kg/m²) compared to the median of the cholecystectomy group (27.9 kg/m²).

Robotic Sigmoidectomies and Anterior Resections

The subgroup analysis for robotic sigmoidectomies and high anterior resections included 22 patients, comprising 10 males (45.5%) and 12 females (54.5%). Among these patients, eight (36.4%) had a history of prior abdominal surgery, potentially increasing procedural complexity. ASA scores indicated a moderate to high-risk cohort, with 13 patients (50.1%) classified as ASA 2 and nine (40.9%) as ASA 3. The median age of the patients was 61.0 years (IQR: 15.0), and the mean BMI was 26.3 kg/m² (SD: 6.2). The vast majority of cases were performed for malignant conditions, with only three cases (13.6%) addressing benign disease (diverticular disease).

**Figure 2:**
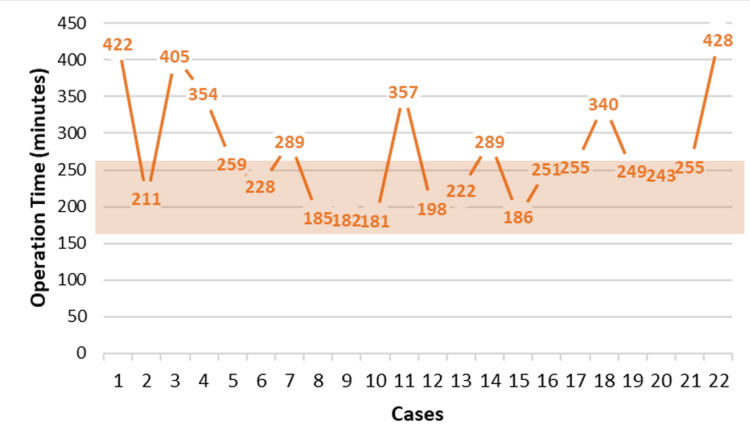
Evolution of intraoperative sigmoidectomy and high anterior resection time The shadowed area represents the average time reported in the literature [7]

Operative characteristics demonstrated a median operative time of 255.0 minutes (IQR: 120.0) (Figure 2). Despite the inherent challenges, there were no conversions to open surgery. The total median length of hospital stay was seven days (IQR: 7.5). Twelve patients required admission to the ICU postoperatively, with a median ICU stay of one day (IQR: 2).

Postoperative outcomes included two readmissions (9.1%), one for small bowel obstruction requiring reoperation (Clavien-Dindo 3b) and another for acute kidney injury secondary to a high-output stoma. Four patients (18.2%) had grade 2 Clavien-Dindo complications, while there was one (4.5%) 30-day mortality in this subgroup, attributed to the patient’s advanced age and increased frailty.

## Discussion

The introduction of RAS into a general surgery department signifies a pivotal evolution in minimally invasive techniques. It demands both skill adaptation and the mastery of innovative technologies. Transitioning from traditional open or laparoscopic to RAS presents a unique learning curve for established consultant surgeons [8]. The move to RAS required comprehensive training, including simulation-based learning, wet-lab practice, observerships, and proctorship, following the Royal College of Surgeons' guidelines [1,9]. RAS has demonstrated significant potential for enhancing minimally invasive surgical care, even within district general hospital settings, as evidenced by our analysis of the first 100 consecutive robotic-assisted general surgery cases at The Hillingdon Hospitals NHS Foundation Trust [10].

Implementing a robotic program within a surgical department offers a range of benefits, such as enhanced precision, superior visualization, and increased dexterity, all of which can lead to improved patient outcomes [11]. Robotic systems enable minimally invasive approaches for complex cases that might otherwise necessitate open surgery, resulting in reduced postoperative pain, less blood loss, and shorter hospital stays [12,13]. For surgeons, the ergonomic design of robotic platforms minimizes fatigue during lengthy procedures and lowers the risk of musculoskeletal strain over time [14]. Additionally, integrating robotics into general surgery promotes a culture of innovation and skill enhancement, attracting top-tier professionals and fostering multidisciplinary collaboration. This environment elevates overall surgical standards, enhances patient satisfaction, and strengthens the hospital’s position as a leader in advanced care. The effectiveness of a new surgical technique can be evaluated through both surgical processes and patient outcomes.

Our results align with and contribute to the existing body of literature on RAS implementation in general surgery [10]. Our patient cohort was not selectively chosen, as we included the first 100 consecutive cases (Table 1); the BMI distribution, with a mean of 27.5 kg/m², was similar to the general population trend toward overweight individuals and reflects findings in robotic surgery studies that demonstrate similar BMI profiles [15]. The median operative times, conversion rates, and complication profiles observed in our cohort are comparable to those reported in other studies on robotic cholecystectomy, hernia repairs, and colorectal resections [7,16-20]. Additionally, our study revealed a notable difference in the median age of patients undergoing minor versus major procedures, with a median age of 50.0 years (IQR: 23.0) for minor operations compared to 69.0 years (IQR: 16.3) for major procedures. This age disparity is consistent with findings indicating that older patients are more frequently selected for complex robotic surgeries due to their potential benefits in reducing surgical morbidity [21]. Despite this age difference, the BMI between the two groups did not differ significantly, with a mean BMI of 27.9 ± 5.2kg/m^2^ for minor procedures and 27 ± 4.8kg/m^2^ for major procedures. This suggests that BMI alone did not influence procedural selection or complexity, as this has already been reported in similar studies [22]. Additionally, 25% of our cohort had prior abdominal surgery, a factor known to increase procedural complexity and pose potential challenges during the robotic learning curve due to adhesions and altered anatomy [23]. 

The operative characteristics of our RAS program encompass a diverse range of procedures. Among the first 100 consecutive cases, cholecystectomies constituted the most common procedure (35 patients). Cholecystectomy is often regarded as the entry-point procedure for general surgeons transitioning to RAS due to its relative simplicity, standardized steps, and the opportunity it provides to develop basic robotic skills [24]. It is important to note that we did not encounter any common bile duct (CBD) injuries or any other major complications, which may be attributed to the adherence to safety protocols (establishing Critical View of Safety), and the structured training program implemented during the transition to robotic surgery [16]. However, it should be mentioned that a recent meta-analysis has reported higher rates of CBD injuries associated with robotic cholecystectomy compared to laparoscopic approaches (0.7% vs 0.2%; relative risk (RR), 3.16 (95% CI, 2.57-3.75)) [3]. 

Specifically, concerning robotic cholecystectomies, the median intraoperative time was 81.0 minutes, which falls within the reported range in the study by Jayaraman et al., who documented the mean robotic cholecystectomy of 91.0 minutes (range: 57.0-167.0 minutes), demonstrating the learning curve and case complexity influence on operative duration. Our cohort included three outliers with operative times exceeding 130 minutes due to difficult cases involving patients with prior abdominal surgery and a BMI over 30. These factors are consistent with the expected limitations and challenges associated with the learning curve of robotic surgery [25].

Furthermore, complex colorectal procedures, such as right colectomies, sigmoidectomies, and low anterior resections, comprised a significant portion of the cases (49 patients), highlighting the ability of RAS to improve precision in oncologic resections and complex anatomical dissections. Among major resections, sigmoidectomy emerged as the most common procedure (22 patients), which is considered one of the more straightforward colonic resections due to its predictable anatomical landmarks and relatively accessible surgical field [26]. The use of RAS in these cases further enhances the precision and efficiency of dissection, particularly in challenging pelvic anatomy, making it an ideal candidate for expanding robotic expertise within general and colorectal surgery practice. Regarding operative time, our cohort demonstrated a median operative time of 255.0 minutes (IQR: 120.0 minutes), which, while comparable, falls at the higher end of the reported range in the literature. Published data suggest that robotic left colectomies and sigmoid resections typically have average operative times ranging from approximately 215.0 minutes (range: 163-267 minutes) [7]. This higher operative time reflects the impact of the learning curve associated with RAS, particularly in complex colorectal procedures, as surgeons acquire proficiency in optimizing robotic technique and workflow.

Postoperative outcomes, including LOS and complication rates, further underscore the safety and efficacy of RAS when integrated into the general hospital settings [20]. As expected, major operations demonstrated a significantly longer LOS, with a total median LOS of six days compared to zero days for minor procedures (p < 0.001), which were mainly ambulatory. Additionally, the ICU admission rate was markedly higher for major procedures at 57.7%, while no ICU admissions were required for minor procedures. This trend reflects the anticipated postoperative demands of complex cases managed with RAS [27]. In terms of reoperation, readmission, and 30-day mortality rates, no statistically significant differences were observed between major and minor procedures, further supporting the safety profile of RAS in both categories. The absence of significant differences in reoperation and readmission rates suggests that RAS may offer consistent and predictable postoperative outcomes, even in complex cases, provided appropriate patient selection and perioperative care protocols are maintained. 

Our zero-conversion rate for minor procedures highlights the ability of RAS to maintain minimally invasive approaches even during the early phases of adoption [3]. In contrast, the 5.8% conversion rate for major procedures reflects the complexity and technical demands of these surgeries, which are more susceptible to conversion during the learning curve [28]. Arquillière et al. reported that after the learning phase consisting of 9-14 robotic-assisted total mesorectal excision cases, the conversion rate decreased significantly [29].

A key strength of this study is its focus on the real-world implementation of RAS within a district general hospital, providing a comprehensive overview of the feasibility, outcomes, and challenges faced during the early stages of adoption. Unlike highly selective or specialized centers, our cohort represents a broad and diverse patient population, ensuring that our findings are applicable to a wide range of general surgery practices. Furthermore, the structured training and proctorship provided to the consultant surgeons ensured a consistent and safe approach to transitioning from laparoscopic to robotic techniques, thereby strengthening the validity of the reported outcomes. 

However, this study has some limitations. First, this is a retrospective study which represents a single-center experience, limiting generalizability to other settings with differing resources and patient demographics. In addition, the analysis focused on the first 100 consecutive cases, capturing a mixed patient population undergoing various types of procedures, contributing to potential selection bias. In addition, although this diversity provides valuable insights, it also introduces variability that may influence outcomes. The variability in our data caused by mixed procedures may have reduced statistical power by increasing error and making it more difficult to detect true differences, and it also complicates subgroup comparisons by introducing potential bias. However, despite these limitations, our a priori aim was to evaluate the RAS program in our hospital and to assess its feasibility and safety. Despite these challenges, our experience underscores that with strategic planning, RAS can be successfully integrated into general surgery practice, offering benefits for the patient, the surgeon, and the overall outcomes.

Looking ahead, further expansion of our RAS program offers promising opportunities to enhance patient care and surgical outcomes. Acquiring a second robotic platform would significantly improve access and scheduling flexibility, thereby optimizing resource utilization and reducing waiting times for complex cases. Additionally, hiring robotic-trained colorectal consultants would bolster surgical expertise and mentorship, fostering skill development across the multidisciplinary team. Beyond colorectal and general surgery, extending the implementation of robotic systems to other subspecialties such as advanced abdominal wall reconstruction, upper gastrointestinal (GI) surgery, bariatrics, and dedicated ambulatory general surgery procedures could unlock additional patient care benefits. This broader integration of RAS will support the hospital's aim to provide comprehensive, state-of-the-art minimally invasive care, further solidifying its role as a leader in robotic surgery within the district general hospital setting.

## Conclusions

This study highlights the successful implementation of RAS in a district general hospital, providing an essential foundation for advancing minimally invasive surgical techniques in such settings. Our evaluation of the first 100 consecutive robotic-assisted general surgery cases demonstrates that RAS provides feasibility, safety, and favorable outcomes, even during the initial adoption phase, with low complication rates, minimal conversions, and comparable results for both minor and major procedures. Strategic investments, such as acquiring additional robotic systems and expanding the pool of trained surgeons, will support the growth and broader application of RAS across subspecialties.
